# Cardioprotective Effect of Whole Body Periodic Acceleration in Dystrophic Phenotype *mdx* Rodent

**DOI:** 10.3389/fphys.2021.658042

**Published:** 2021-05-04

**Authors:** Arkady Uryash, Alfredo Mijares, Eric Esteve, Jose A. Adams, Jose R. Lopez

**Affiliations:** ^1^Division of Neonatology, Mount Sinai Medical Center, Miami Beach, FL, United States; ^2^Centro de Biofísica y Bioquímica, Instituto Venezolano de Investigaciones Científicas, Caracas, Venezuela; ^3^UMR 5525 UGA-CNRS-Grenoble INP-VetAgro Sup TIMC, Université Grenoble Alpes, Grenoble, France; ^4^Department of Molecular Biosciences, University of California, Davis, Davis, CA, United States; ^5^Department of Research, Mount Sinai Medical Center, Miami Beach, FL, United States

**Keywords:** Duchenne cardiomyopathy, calcium, oxidative stress, cardiac contractility, utrophin, l-name

## Abstract

Duchenne muscular dystrophy (DMD) is characterized by progressive muscle wasting and the development of a dilated cardiomyopathy (DCM), which is the leading cause of death in DMD patients. Despite knowing the cause of DMD, there are currently no therapies which can prevent or reverse its inevitable progression. We have used whole body periodic acceleration (WBPA) as a novel tool to enhance intracellular constitutive nitric oxide (NO) production. WBPA adds small pulses to the circulation to increase pulsatile shear stress, thereby upregulating endothelial nitric oxide synthase (eNOS) and neuronal nitric oxide synthase (nNOS) and subsequently elevating the production of NO. Myocardial cells from dystrophin-deficient 15-month old *mdx* mice have contractile deficiency, which is associated with elevated concentrations of diastolic Ca^2+^ ([Ca^2+^]_d_), Na^+^ ([Na^+^]_d_), and reactive oxygen species (ROS), increased cell injury, and decreased cell viability. Treating 12-month old *mdx* mice with WBPA for 3 months reduced cardiomyocyte [Ca^2+^]_d_ and [Na^+^]_d_ overload, decreased ROS production, and upregulated expression of the protein utrophin resulting in increased cell viability, reduced cardiomyocyte damage, and improved contractile function compared to untreated *mdx* mice.

## Introduction

Duchenne muscular dystrophy (DMD) is caused by the lack of expression of dystrophin, which forms part of the dystroglycan structural complex in the sarcolemma (Hoffman et al., 1987; Muntoni et al., 2003). The absence of dystrophin produces a dysfunctional regulation of intracellular Ca^2+^ in skeletal muscle of DMD patients (Lopez et al., 1987) and in dystrophin-deficient *mdx* mice, one of the most frequently used animal models of DMD (Allen et al., 2010a; Altamirano et al., 2014) as well as in cardiac muscle (Mijares et al., 2014; Lopez et al., 2017). Furthermore, a disturbed intracellular Ca^2+^ regulation has also been found in smooth muscle (Lopez et al., 2020) and neurons (Lopez et al., 2016) from dystrophic mice. In addition to their progressive debilitating skeletal muscle myopathy, DMD patients as they aged develop a lethal dilated cardiomyopathy (DCM) with arrhythmias, myocardial fibrosis, and congestive heart failure (Sanyal and Johnson, 1982; Kamdar and Garry, 2016; Mavrogeni et al., 2018), which is the leading cause of death in DMD patients (Mazur et al., 2012; Passamano et al., 2012).

Presently, there are no effective treatments for DMD. Although various therapeutic approaches have been developed, they have not had a meaningful impact on DMD patients’ long-term health (Schinkel et al., 2012; Zhang and Duan, 2012) or unexpectedly have aggravated the cardiomyopathy in those patients (Townsend et al., 2009). Corticosteroid therapy still represents the primary treatment option, but it has serious side effects, limited skeletal muscle improvement, and it has and few to no benefits in preventing or treating DMD cardiomyopathy (Malik et al., 2012; Bylo et al., 2020).

Alternative therapies to treat DMD have been proposed in animal models based on enhancing nitric oxide (NO) production by providing NO-substrate availability like but (Ramachandran et al., 2013; Vianello et al., 2013), using NO donors (Marques et al., 2005; Miglietta et al., 2015), phosphodiesterase inhibitors (Percival et al., 2012; de Arcangelis et al., 2016), however, the outcome from human clinical trials have shown no effect (Victor et al., 2017) or a modest patient improvement (Nelson et al., 2014; Hafner et al., 2019). Whole body periodic acceleration (WBPA) is a non-invasive and non-pharmacological approach that increases pulsatile shear stress to the vascular endothelium causing upregulation of the expression of both endothelial NO synthase (eNOS) and neuronal NO synthase (nNOS) and the subsequent release of NO in humans (Fujita et al., 2005; Sackner et al., 2005a, b) and animal models (Adams et al., 2009; Uryash et al., 2009; Wu et al., 2009; Altamirano et al., 2014). We have previously demonstrated that WBPA is an effective therapy alleviating the alterations observed in skeletal, cardiac muscles, and cortical neurons from young dystrophin-deficient *mdx* and dystrophin/utrophin double knock out mice (Altamirano et al., 2014; Lopez et al., 2017, 2018).

This study we tested the hypothesis that WBPA can ameliorate or reverse the severity of cardiomyopathy in dystrophin-deficient *mdx* mice by reducing diastolic [Ca^2+^] and [Na^+^] and oxidative stress and upregulating utrophin expression, which leads to a reduction in cardiomyocytes damage, increased cell viability, and improved cardiomyocyte contractility. We provide evidence that a mechanism that is related to NO mediates the WBPA-provoked cardioprotection. Our results raise the hope for lessening dystrophic cardiomyopathy in humans by enhancing NO signaling using WBPA.

## Materials and Methods

### Animal Model

WT (C57BL10) and dystrophic *mdx* (C57BL/10ScSn-Dmd^*mdx*^) 12-month old (mos) mice (middle-aged) were obtained from breeding colonies at the Mount Sinai Medical Center were used as experimental models. Founders for these colonies were initially obtained from The Jackson Laboratory (Bar Harbor, ME, United States). All animals were housed under constant temperature (21–22°C) and humidity (50–60%) with a 12:12 light-dark cycle and provided food and water *ad libitum.* 12 old-month *mdx* mice were used because changes in heart function appear to be well established at that time, as well as in older *mdx* mice (Adamo et al., 2010). Male WT (*N* = 32) and *mdx* (*N* = 89) mice 12-month of age were randomly assigned to four groups: (a) WT, untreated; (b) WT, treated with WBPA; (c) *mdx*, untreated; (d) *mdx*, treated with WBPA.

### Cardiomyocyte’s Isolation and Inclusion Criteria

With the animal under deep anesthesia (isoflurane), thoracotomy was performed, heart was excised, and LV myocytes were enzymatically dissociated from WT and *mdx* mice 15 months WBPA-treated and untreated mice using the method described previously (Liao and Jain, 2007). All cardiomyocytes used in this study met the following criteria: (i) a well-defined striation spacing with good edges; (ii) no spontaneous contraction when perfused with a physiological solution containing 1.8 mM Ca^2+^; and (iii) Resting sarcomere length ≥1.75 μm, determined at the beginning of the experiment (Wussling et al., 1987).

### Whole Body Periodic Acceleration Protocol

Awake unanesthetized mice placed in a rodent holder (Kent Scientific, CT, United States) positioned on a motion platform (SK-L180-Pro, Scilogex, CT, United States) at a frequency of 480 cycles per min (cpm) for 1 h daily, 5 days a week, for 12 consecutive weeks. Untreated mice were placed in the rodent holder for 1 h daily, 5 days a week, for 12 consecutive weeks without receiving WBPA treatment.

### Preparation of Ion-Selective Microelectrodes

Double-barreled Ca^2+^-selective microelectrodes were prepared and backfilled with the neutral carrier ETH-129 (21193 Sigma-Aldrich, St. Louis, MO, United States) and then with pCa 7 solution as described previously (Lopez et al., 2000; Eltit et al., 2013). Double-barreled Na^+^-selective microelectrodes were prepared as above but backfilled with the Na^+^-sensitive ion cocktail (Sodium Ionophore I Cocktail A, Sigma-Aldrich, St. Louis, MO, United States) based on the neutral ligand ETH-227, as described previously (Eltit et al., 2013). The characteristics and properties of the ion-selective microelectrodes were similar to those described previously (Lopez et al., 1983, 2000; Eltit et al., 2013). After taking measurements of diastolic calcium concentration ([Ca^2+^]_d_) or diastolic sodium concentration ([Na^+^]_d_), all microelectrodes were recalibrated, and if the two calibration curves did not agree within 3 mV, data was discarded.

### Recording of [Ca^2+^]_d_ and [Na^+^]_d_

Isolated cardiomyocytes from WBPA-treated and untreated mice were impaled with either Ca^2+^ or Na^+^ double-barreled selective microelectrodes. The potentials from 3 M KCl microelectrode barrel (resting membrane potential – Vm) and Ca^2+^microelectrode barrel (V_*Ca*_) or from V_*m*_ and Na^+^ microelectrode barrel (V_*Na*_) were recorded via high impedance amplifier (WPI, DUO-223 electrometer, Sarasota, FL, United States) (Mijares et al., 2014; Lopez et al., 2017). The potential from the V_*m*_ barrel was subtracted electronically from the V_*Ca*_ or V_*Na*_ potential to produce a differential Ca^2^ potential (V_*Diff*__*E*_) or Na^+^ (V_*Diff*__*E*_) potential that represents the cell [Ca^2+^]_d_ or [Na^+^]_d_. The potentials recorded were stored in a computer for further analysis. Experiments were carried at 37°C.

### Measurement of Intracellular ROS

Intracellular ROS accumulation was measured in cardiomyocytes from WBPA-treated and untreated mice using 2,7-dichlorodihydrofluorescein diacetate according to the manufacturer’s recommendation (DCDHF-DA, Abcam, MA, United States). The DCF fluorescence was detected by a microplate reader (Molecular Device, Sunnyvale CA, United States) at an excitation of 488/20 nm and 525/540 nm emission wavelengths. Intensity values are reported normalized to untreated WT values after subtracting background fluorescence.

### Cardiomyocyte Damage

Myocardial injury was determined in WBPA-treated and untreated mice by measuring plasma levels of cardiac troponin T (cTnT) quantified by ELISA (Boehringer Mannheim, Indianapolis, IN, United States) following the manufacturer’s recommendations. Blood samples were drawn before and after WBPA or control treatment from the tail vein.

### Cardiomyocyte Viability

Cardiomyocyte viability from WBPA-treated and untreated 15 months-old mice was determined using the 3-(4,5-dimethylthiazol-2-yl)-2,5-diphenyltetrazolium bromide (MTT) assay according to the manufacturer’s protocol (Abcam, MA, United States). Data collected from WBPA-treated and untreated *mdx* and WT mice are represented as a reduction in MTT concentration relative to untreated WT cardiomyocytes.

### Resting Sarcomere Length

Resting sarcomere length was determined in quiescent cardiomyocytes isolated from 15-month old mice after a 15-s stimulation through a pair of platinum electrodes (1 Hz, 1 ms pulse duration, ∼1.5x threshold voltage) at 37°C using the video-edge detection system (IonOptix, Milton, MA, United States) (Lopez et al., 2017).

### Measurement of Myocyte Contractility

We carried out contractile studies on isolated cardiac myocytes from WBPA-treated and untreated mice using a video-based edge-detection system (IonOptix, Milton, MA, United States), as previously described (Lopez et al., 2017). The cardiomyocytes were field stimulated through a pair of platinum electrodes at a frequency of 2 Hz (2 ms pulse duration, ∼1.5x threshold voltage) at 37°C. The following indices were determined: (i) peak shortening (PS), (ii) maximal velocity of shortening (+dL/dt), (iii) maximal velocity of relengthening (-dL/dt).

### Western Blotting of Cardiac Muscle

Another cohort of 15-month WBPA-treated (*N* = 3) and untreated (*N* = 3) dystrophin-deficient *mdx* mice were euthanized using CO_2_. Ventricular tissues were dissected, minced, and homogenized in modified radioimmunoassay precipitation assay (RIPA) buffer as described before (Altamirano et al., 2014). Protein concentration was determined using the Pierce BCA Protein Assay Kit (Thermo Fisher Scientific, MA, United States). Proteins were separated on SDS gel, transferred to a nitrocellulose membrane, and incubated with primary. Equal amounts of total protein were separated on 4–12% NuPAGE Novex Bis-Tris SDS-PAGE Gels (Invitrogen Corporation, Carlsbad, CA, United States) and transferred to Immobilon-FL PVDF membrane (Millipore Corporation, Billerica, MA, United States). The following primary antibodies were used; dystrophin and utrophin and then secondary fluorescent antibodies (Abcam, MA, United States). Glyceraldehyde 3-phosphate dehydrogenase (Abcam, Cambridge, MA, United States) was used as protein loading controls. Blots were visualized by Enhanced Chemifluorescence (ECF) (GE Healthcare Bio-Sciences Corporation, Piscataway, NJ, United States) on Storm 860 Imaging System (GE Healthcare Bio-Sciences Corporation, Piscataway, NJ, United States).

### L-NAME Pretreatment

L-NAME is a non-selective inhibitor of NO-synthase used to induce NO synthesis deficiency (Furfine et al., 1997). In a different cohort of 12-month dystrophin-deficient *mdx* mice were then randomized in four groups: (A) *mdx* no treatment (*N* = 7 mice); (B) WBPA-treated *mdx* for 12 weeks (*N* = 7 mice); (C) *mdx* mice pre-treated with L-NAME for 1 week before and throughout the WBPA treatment protocol (12 weeks) (*N* = 9 mice); (D) *mdx* mice pre-treated with L-NAME for 13 weeks (*N* = 7 mice). L-NAME (1 g/L) was provided in drinking water (*ad libitum*), equivalent to a dose of 180 mg/kg. This L-NAME dose inhibits NOS activity and can be administered for prolonged periods (Lampson et al., 2012). The water was replaced three times per week with fresh L-NAME containing water, and all L-NAME treated mice were placed in individual cages to evaluate daily water intake. Upon completing the protocol, [Ca^2+^]_d_ and intracellular ROS production were measured in cardiomyocytes from all four groups as markers of cardioprotection.

### Solutions

Normal Tyrode solution was made up as follows (in mM): 140 NaCl, 2.5 KCl, 1.8 CaCl_2_, 1 MgCl_2_, 5 HEPES, 10 glucose (pH 7.4). The Tyrode solution was continuously bubbled with 95% O_2_ and 5% CO_2_ gas mixture.

### Statistical Analysis

No explicit power calculation was conducted before experimentation; the sample sizes used were based on our previous studies using WT and dystrophic mouse models (Mijares et al., 2014; Lopez et al., 2017). For multiple groups comparison, a one-way analysis of variance (ANOVA) test was used, followed by Tukey’s tests and student *t*-test for the Western blotting experiments to determine the degree of significance. Reported results are presented as mean ± S.E.M., with *N* representing the number of mice used and *n* representing the number of cardiomyocytes in which measurements were carried out. GraphPad Prism 9 (GraphPad Software Inc., La Jolla, CA, United States) was used for statistical analyses. A *p-*value < 0.05 was considered statistically significant (^∗^*p* < 0.05, ^∗∗^*p* < 0.01, and ^∗∗∗^*p* < 0.001).

## Results

### WBPA Normalized Aberrant [Ca^2+^]_d_ and [Na^+^]_d_ in *mdx* Cardiomyocytes

**FIGURE 1:**
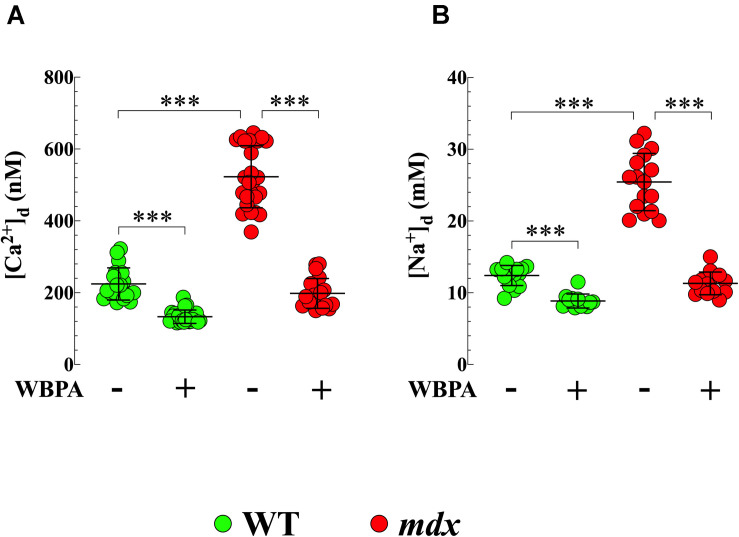
Whole body periodic acceleration lowered [Ca^2+^]_d_ and [Na^+^]_d_ in cardiomyocytes. [Ca^2+^]_d_ and [Na^+^]_d_ was measured in cardiomyocytes from 15-month treated and untreated, WT and *mdx* mice using ion-specific microelectrodes. Diastolic [Ca^2+^] **(A)** and [Na^+^] **(B)** were significantly higher in *mdx* than WT cardiomyocytes (2.4-fold and 2-fold, respectively). Whole body periodic acceleration (WBPA) reduced [Ca^2+^]_d_ by 2.6-fold in *mdx* cardiomyocytes and by 1.7-fold in WT cardiomyocytes **(A)**, while that [Na^+^]_d_ was reduced by 2.2-fold in *mdx* cardiomyocytes and by 1.4-fold in WT cardiomyocytes **(B)**. [Ca^2+^]_d_ measurements: Untreated and WBPA-treated WT, *N* = 3 mice per experimental condition, *n* = 19–21, respectively; Untreated and WBPA-treated *mdx N* = 4 mice, *n* = 25–19, respectively. [Na^+^]_d_ measurements: Untreated and WBPA-treated WT, *N* = 3 mice per experimental conditions, *n* = 15–12, respectively; Untreated and WBPA-treated *mdx N* = 3 per experimental condition, *n* = 16–14, respectively. Values are expressed as means ± S.D. One-way ANOVA with Tukey’s post-test, ***p ≤ 0.001.

[Ca^2+^]_d_ in cardiomyocytes from WT 15-month mice was 224 ± 45 nM while that in *mdx* cardiomyocytes was 523 ± 87 nM (*p* < 0.001 compared to WT) (Figure 1A). Treatment with WBPA significantly reduced [Ca^2+^]_d_ in WT cardiomyocytes to 133 ± 19 nM, levels similar to those observed in young WT mice (3-month of age) (Mijares et al., 2014) (*p* < 0.001 compared to non-WBPA-treated WT) and in *mdx* cells from 523 ± 88 to 198 ± 42 nM (*p* < 0.001 compared to non-WBPA-treated *mdx* and *p* > 0.05 compared to non-WBPA-treated WT) (Figure 1A).

We also found that [Na^+^]_d_ was more elevated in cardiomyocytes from dystrophin-deficient *mdx* 15-month mice (25 ± 4 mM) compared to WT cardiomyocytes (12 ± 1.2 mM, *p* < 0.001) (Figure 1B). WBPA treatment significantly lowered [Na^+^]_d_ in WT cardiomyocytes to 8.7 ± 1 mM, intracellular concentration comparable to those observed in young WT mice (Mijares et al., 2014) (*p* < 0.001 compared to non-WBPA-treated WT) and to 11 ± 2 mM in *mdx* cardiomyocytes (*p* < 0.001 compared to non-WBPA-treated *mdx* and *p* > 0.05 compared to non-WBPA-treated WT) (Figure 1B). These results demonstrate that WBPA treatment reduced cardiomyocytes Ca^2+^ and Na^+^ overload found in 15-month WT and *mdx* mice.

### WBPA Decreased Formation of Reactive Oxygen Species in *mdx* Cardiomyocytes

**FIGURE 2:**
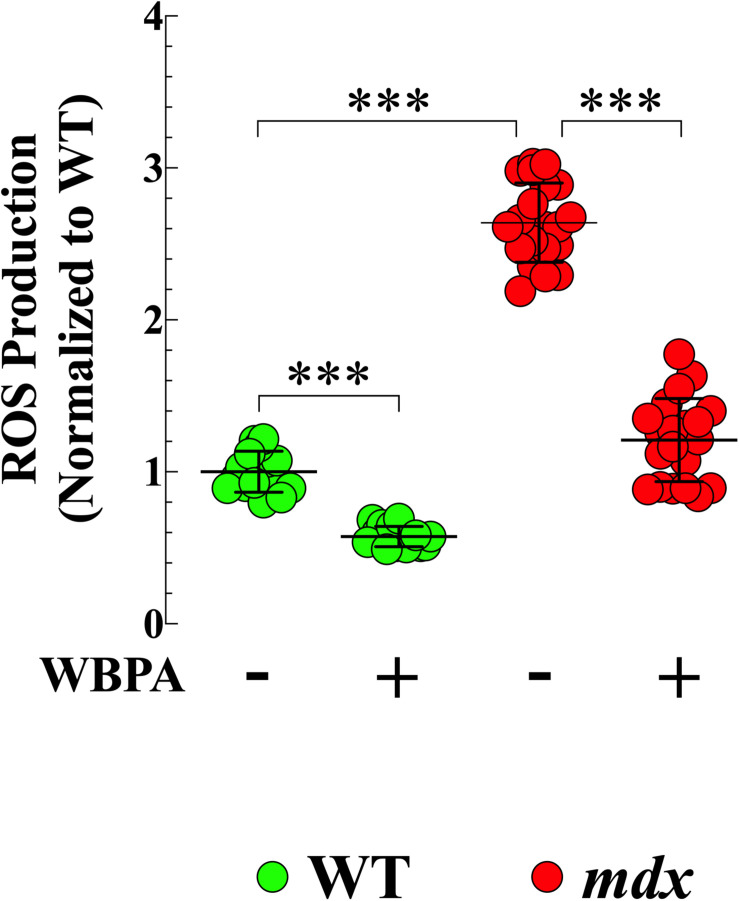
Whole body periodic acceleration reduced ROS generation in cardiomyocytes. Cardiomyocytes ROS production was evaluated in 15-month WBPA-treated and untreated WT and *mdx* dystrophin-deficient mice. Intracellular ROS was 2.6-fold higher in *mdx* cardiomyocytes than WT. WBPA treatment reduced intracellular ROS production by 2.7-fold in *mdx* cardiomyocytes and by 1.7-fold in WT cardiomyocytes compared to untreated *mdx* and WT cardiomyocytes. *ROS measurements*: Untreated and WBPA-treated WT, *N* = 3 mice per experimental conditions, *n* = 13–15, respectively. Untreated and WBPA-treated *mdx N* = 3 mice per experimental conditions, *n* = 17–20, respectively. Values are expressed as means ± S.D. One-way ANOVA with Tukey’s post-test, ***p ≤ 0.001.

Oxidative stress has been suggested is to be an important contributing factor to the muscle damage and weakness inherent to muscular dystrophy (Rodriguez and Tarnopolsky, 2003; Kaczor et al., 2007). We have shown previously that ROS production is elevated in young cardiomyocytes from mice lacking both dystrophin and utrophin and that WBPA partially improves intracellular ROS dyshomeostasis in those cells (Lopez et al., 2017). We found 2.6-fold increased production of ROS in 15-month *mdx* cardiomyocytes compared to WT cardiomyocytes (Figure 2). WBPA treatment reduced the levels of ROS in WT cardiomyocytes by 1.7-fold (*p* < 0.001) and in *mdx* cardiomyocytes by 2.7-fold (*p* < 0.001 compared to WBPA-untreated cardiomyocytes (Figure 2).

### WBPA Promoted Upregulation of Utrophin

**FIGURE 3:**
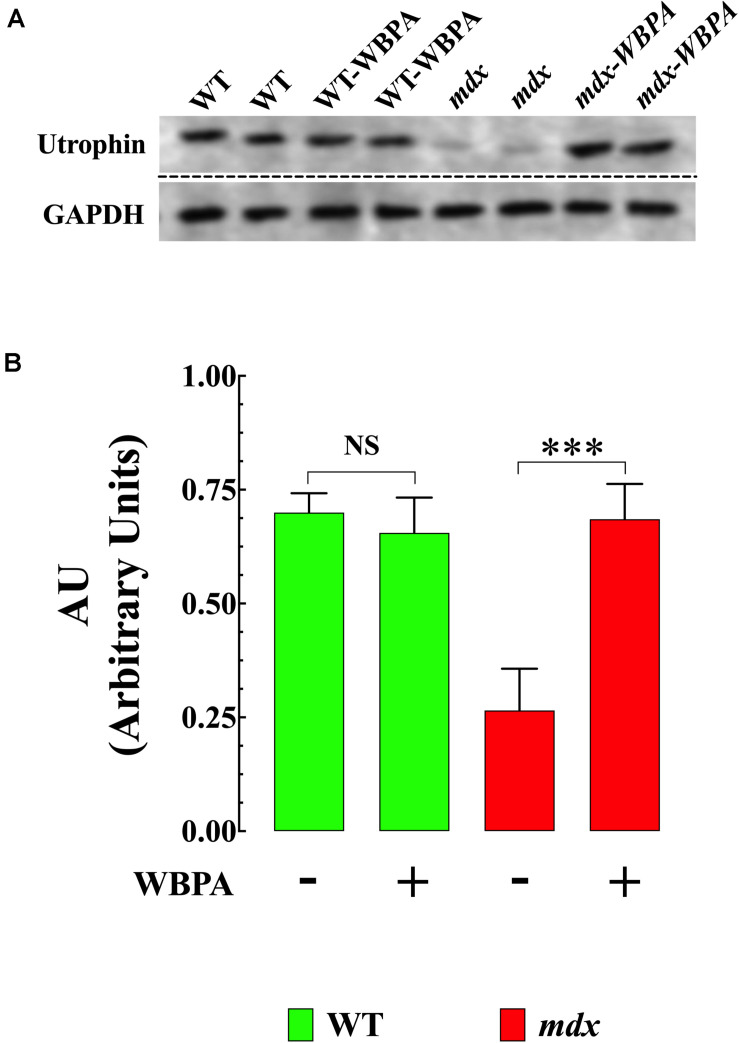
Whole body periodic acceleration enhanced expression of utrophin in *mdx* heart. **(A)** Representative Western blot showing the increase in utrophin expression in WBPA-treated *mdx* dystrophin-deficient heart homogenates compared to heart homogenates from untreated *mdx* mice. WBPA did not modify utrophin expression in WT. **(B)**. Illustrated the densitometric analysis of Western blots proteins bands from WBPA-treated and untreated *mdx* heart. Data were normalized to GAPDH and expressed as mean ± S.D. Untreated *mdx N* = 3 mice and WBPA-treated *mdx N* = 3 mice. Paired *t*-test, ***p ≤ 0.001.

Previous studies have demonstrated that upregulation of utrophin (UTR) in dystrophin-deficient *mdx* mice results in the amelioration of pathology (Tinsley et al., 1998; Chaubourt et al., 1999; Cerletti et al., 2003). WBPA significantly upregulated utrophin expression (1.5-fold) in *mdx* heart compared to untreated *mdx* mice (Figures 3A,B). No significant effect was observed in WT cardiomyocytes.

### Attenuation of *mdx* Cardiomyocyte Damage by WBPA

**FIGURE 4:**
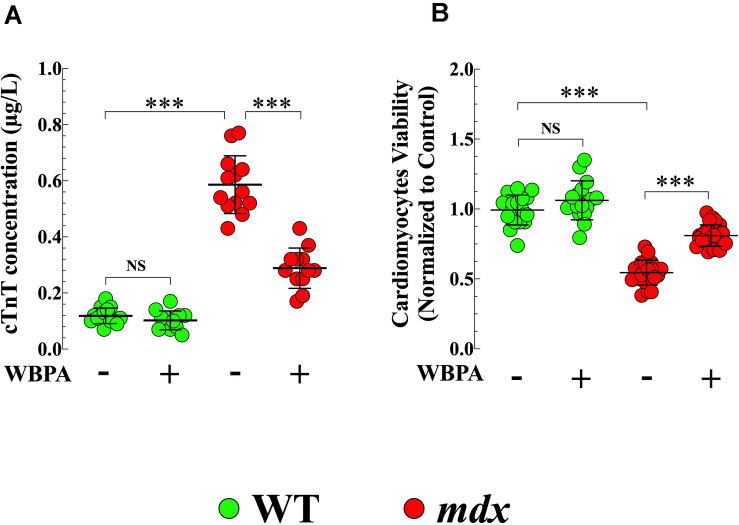
Whole body periodic acceleration reduced cTnT plasma level and increased cell viability. Measurements cTnT plasma level **(A)**, and cardiomyocyte viability **(B)** were evaluated in 15-month WBPA-treated and untreated WT and *mdx* dystrophin-deficient mice. cTnT plasma level was 4.9-fold more elevated **(A)** in *mdx* than WT cardiomyocytes. Furthermore, *mdx* cardiomyocytes showed a reduction in cell viability by 1.7-fold compared to WT cardiomyocytes **(B)**. WBPA lower cTnT plasma level by 2-fold **(A)**, and increased cell viability by 1.4 compared to WBPA-untreated *mdx* **(B)**. No effect of WBPA on cTnT release and cell viability was observed in WT cardiomyocytes **(A,B)**. *cTnT plasma level*: Untreated and WBPA-treated WT, *N* = 5 mice per experimental conditions, *n* = 15–12, respectively; Untreated and WBPA-treated *mdx N* = 5 mice, per experimental conditions *n* = 13–12, respectively. *Cell viability*: Untreated and WBPA-treated WT, *N* = 3 mice per experimental conditions, *n* = 18–16, respectively; Untreated and WBPA-treated *mdx N* = 5 mice, *n* = 20–24, respectively. Values are expressed as means ± S.D. One-way ANOVA with Tukey’s post-test, ***p ≤ 0.001.

Cardiac troponin T (cTnT) is an established blood biomarker with high sensitivity and specificity for myocardial injury (Wu et al., 2017). Plasma cTnT concentrations were significantly higher (4.9-fold, *p* < 0.001) in untreated *mdx* mice compared to WT (Figure 4A). WBPA treatment significantly reduced cTnT level by 2-fold (*p* < 0.001) in *mdx* mice, but it did not modify plasma levels of cTnT in WT mice (*p* = 0.89) (Figure 4A).

### WBPA Improved *mdx* Cardiomyocytes Viability

In the heart, the absence of dystrophin causes cardiac muscle degeneration that compromises the integrity of the sarcolemma, resulting in progressive and irreversible cardiomyocyte degeneration that would induce cell death (Kamogawa et al., 2001). Untreated *mdx* cardiomyocytes showed 1.8-fold less viability compared to WT cardiomyocytes (*p* < 0.001) (Figure 4B). Treatment with WBPA increased the viability of *mdx* cardiomyocytes by 1.5-fold compared to untreated *mdx* cardiomyocytes (Figure 4B). Although WBPA increased viability slightly in WT cardiomyocytes, the difference was not significant (*p* = 0.161).

### Effects of WBPA on the Reduced Contractility in *mdx* Cardiomyocytes

**FIGURE 5:**
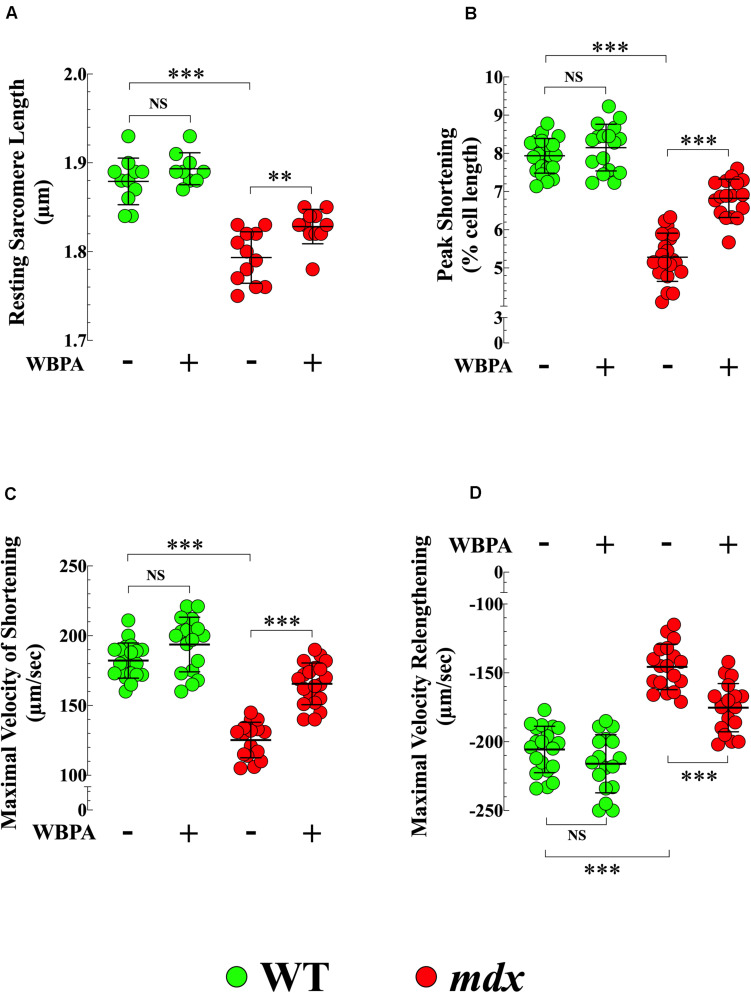
Whole periodic body acceleration improved reduced contractility of *mdx* cardiomyocytes. Contractility was studied in cardiomyocytes from 15-month treated and untreated, WT and *mdx*. The resting sarcomere length was smaller in dystrophin-deficient *mdx* cardiomyocytes compared to WT **(A)**. *Mdx* cardiomyocytes showed a decrease of peak shortening (PS) **(B)**, of maximal velocity of shortening (+dL/dt) **(C)**, and of maximal velocity of relenthening (–dL/dt) **(D)** compared with age-match WT. The WBPA treatment returned the diastolic sarcomere length of *mdx* near WT cardiomyocytes values **(A)** and partially reversed the contractile dysfunction by significantly increasing PS **(B)**, +dL/dt **(C)**, and –dL/dt **(D)**. WBPA treatment did not significantly modify the contractile properties in WT cardiomyocytes, **(C)**. Untreated and WBPA-treated WT, *N* = 4 mice per experimental conditions, *n* = 21–17, respectively; Untreated and WBPA-treated *mdx, N* = 6 mice, per experimental conditions *n* = 17–20, respectively. Values are expressed as means ± S.D. One-way ANOVA with Tukey’s post-test, **p ≤ 0.01, ***p ≤ 0.001.

Cardiac contractile dysfunction is the leading cause of morbidity and mortality in DMD patients (Kamdar and Garry, 2016; Fayssoil et al., 2017). We found that the average diastolic sarcomere length was significantly different between 15-month WT and *mdx* cardiomyocytes (1.87 ± 0.01 vs. 1.79 ± 0.02 μm, *p* < 0.001) (Figure 5A), and that *mdx* cardiomyocytes exhibited a 1.5-fold decrease in peak shortening (PS) (*p* < 0.001), 1.5-fold decrease in maximal velocity of shortening (+dL/dt) (*p* < 0.001), and 1.4-fold decrease in the maximal velocity of relengthening (−dL/dt) (*p* < 0.001), compared with WT (Figures 5B–D). The WBPA treatment returned the diastolic sarcomere length of *mdx* cardiomyocytes to near WT values (1.82 ± 0.01 μm, *p* < 0.05) (Figure 5A) and significantly increased PS and +dL/dt by 1.3-fold (*p* < 0.001 and increased −dL/dt by 1.1-fold (*p* < 0.01) (Figures 5B–D). WBPA treatment did not significantly modify the contractile functions in WT cardiomyocytes, with the exception that +dL/dt that increased significantly (*p* < 0.03).

### Involvement of NO in the Cardioprotective Effects of WBPA

**FIGURE 6:**
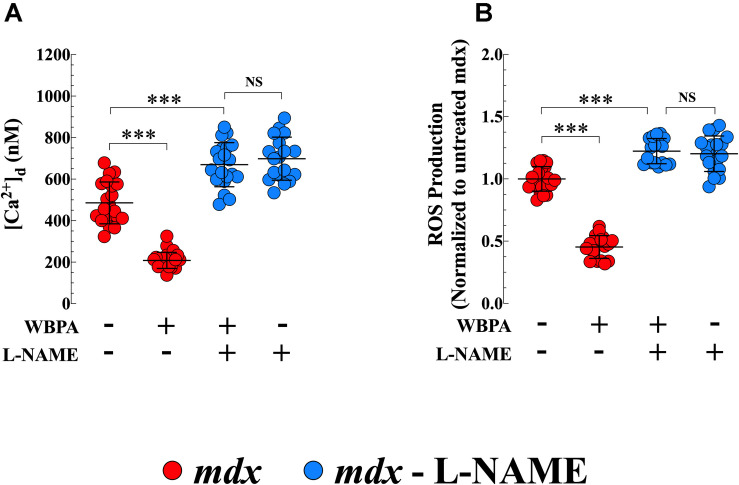
L-NAME pretreatment abolished the cardioprotection induced by WBPA. Pretreatment of dystrophin-deficient *mdx* mice with L-NAME abrogated the beneficiary effect of WBPA on diastolic [Ca^2+^] **(A)** and intracellular ROS production **(B)**. Interestingly, L-NAME pretreatment aggravated the [Ca^2+^]_d_ overload and oxidative stress in *mdx* cardiomyocytes **(A,B)**. *[Ca^2+^]_d_ measurements*: Untreated and WBPA-treated *mdx*, *N* = 4 mice per experimental condition, *n* = 21–24, respectively; L-NAME and WBPA pretreated *mdx N* = 5 mice, *n* = 20; L-NAME pretreated *mdx N* = 4 mice, *n* = 18. *ROS measurements*: Untreated and WBPA-treated *mdx*, *N* = 3 mice per experimental conditions, *n* = 20–18, respectively; L-NAME and WBPA pretreated *mdx N* = 4 mice, *n* = 17; L-NAME pretreated *mdx N* = 3 mice, *n* = 17. Values are expressed as means ± S.D. One-way ANOVA with Tukey’s post-test, ***p ≤ 0.001.

L-NAME is widely used as an inhibitor of NOS activity (Palmer and Moncada, 1989). Pretreatment of *mdx* mice with L-NAME abolished the effects of WBPA on [Ca^2+^]_d_ and intracellular ROS production in *mdx* cardiomyocytes (Figures 6A,B). Interestingly, inhibition of NOS synthase by L-NAME in *mdx* mice further increased cardiomyocyte [Ca^2+^]_d_ by 1.4-fold and ROS generation by 1.2-fold compared to untreated *mdx* mice (Figures 6A,B).

## Discussion

Our results demonstrate that WBPA treatment provides cardioprotection and improves myocyte function in dystrophin-deficient *mdx* mice with established cardiomyopathy by reducing intracellular Ca^2+^ and Na^+^ overload, reducing intracellular ROS generation, enhancing the expression of utrophin, reducing cardiomyocyte damage, with the end result of improvement of cell viability and contractile function. Mechanistically, we provided evidence that the effect of WBPA on the heart is mediated through the nitric oxide pathway.

### Cellular Dysfunction in *mdx* Cardiomyocytes From Mice With a Proven Cardiomyopathy

Duchenne muscular dystrophy (DMD) is a degenerative disorder of striated and smooth muscle cells, characterized by the absence of the cytoskeletal protein dystrophin (Hoffman et al., 1987; Allen et al., 2010b). DMD patients show a dilated cardiomyopathy leading to heart failure, which is now the leading cause of mortality in DMD patients (Passamano et al., 2012). The early onset of dystrophic dilated cardiomyopathy (DCM) is usually underdiagnosed because most dystrophic patients are asymptomatic in the disease’s initial phases. The development of DCM results from numerous mechanisms, one of which is an increase in diastolic [Ca^2+^] as seen in *mdx*, a mouse model of DMD (Williams and Allen, 2007a; Mijares et al., 2014; Lopez et al., 2017). The underlying mechanisms that led to this increase in [Ca^2+^]_d_ are not yet fully understood; however, evidence points to S-nitrosylation of type 2 ryanodine receptor (RyR2) (Bellinger et al., 2009; Wang et al., 2015), resulting in SR-Ca^2+^ leak (Fauconnier et al., 2010), which contributes to the diastolic Ca^2+^ dysregulation observed in dystrophic cardiomyocytes. Furthermore, S-nitrosylation of transient receptor potential canonical (TRPC) channels (Chung et al., 2017) may enhance the Ca^2+^ influx provoking a further elevation of intracellular [Ca^2+^] as seen in *mdx* smooth muscle cells (Lopez et al., 2020). In addition, an increased Na^+^/Ca^2+^ exchanger function (Williams and Allen, 2007a), and ROS production (Williams and Allen, 2007b; Lopez et al., 2017) may also contribute to the intracellular calcium dyshomeostasis. Congruent with our previous reports (Mijares et al., 2014), we have confirmed that the lack of dystrophin in *mdx* cardiomyocytes with proven cardiomyopathy causes an age-dependent [Ca^2+^]_d_ and [Na^+^]_d_ overload (15-month > 12-month > 3-month) compared with age-matched WT cardiomyocytes. Abnormal intracellular Ca^2+^ regulation appears to play a major role in the molecular pathogenesis of DMD cardiomyopathy.

Reactive oxygen species are critical intracellular signaling molecules that control cellular metabolism, cell proliferation, cellular migration, cell death, and other cellular functions (Holmstrom and Finkel, 2014). Oxidative stress plays an important role in the pathogenesis of several cardiovascular pathologies, such as cardiac hypertrophy, heart failure, myocardial infarction, hypertension (Paravicini and Touyz, 2008; Nabeebaccus et al., 2011), and DCM (Williams and Allen, 2007b). Oxidation of the intracellular environment appears to initiate post-translational modifications of RyR2 channels in the sarcoplasmic reticulum and TRPC channels at the sarcolemma leading to an aberrant intracellular Ca^2+^, which results in activation of apoptotic and necrotic pathways and consequently, the loss of cardiomyocytes functions. We have found intracellular ROS production is significantly increased in *mdx* cardiomyocytes compared to WT. Exaggerated basal rate of ROS generation in dystrophic skeletal muscle is associated with protein oxidation causing inflammation and fibrosis (Valen et al., 2001), mitochondria damage (Jung et al., 2008), and sarco-endoplasmic reticulum stress (Bravo-Sagua et al., 2020), which further aggravates the already substantial diastolic Ca^2+^ overload. There is a close connection between intracellular Ca^2+^ homeostasis and oxidative stress, and increasing evidence suggests close crosstalk between Ca^2+^ and ROS signaling systems, which fine-tunes cellular signaling networks (Gorlach et al., 2015). Thus, disruption of one system can severely impact the other, triggering a cascade of pathological signaling events, ultimately causing dysfunction, disease, or cell death. The mechanism by which high ROS production increases [Ca^2+^] is not fully understood; however, it seems to be mediated by augmented RyR leak and activation of plasmalemmal Ca^2+^ influx (Di et al., 2016). On the other hand, primary elevation of intracellular Ca^2+^ through RyR leak and/or an enhanced Ca^2+^ influx triggers ROS production by uncoupling mitochondrial function, reducing mitochondrial membrane potential (Hsu et al., 2016). The accumulated evidence suggests that the Ca^2+^ and ROS signaling pathways exhibit mutual and synergistic crosstalk with each other. Further experiments in *mdx* cardiomyocytes would be needed to corroborate this hypothesis.

The absence of dystrophin in *mdx* mice alters the dystrophin-associated protein complex making the plasma membrane more fragile to mechanical stress (Yasuda et al., 2005). The increased membrane fragility causes reduced cell viability (Lopez et al., 2017), which was demonstrated here both by reduced cardiomyocyte viability and elevated plasma cTNT levels.

The lack of dystrophin in the *mdx* mouse provokes the develops cardiac abnormalities causing progressive diastolic and systolic dysfunction (Zhang et al., 2008; Li et al., 2009) and subsequent cardiac contractile dysfunction (Sapp et al., 1996). In the present study, we found that *mdx* mice show a reduced contractile response compared to WT. The abnormal contractile response was manifested by a reduced peak shortening, maximal velocity of shortening, and relengthening. It had been shown that force amplitudes were markedly depressed in atrial and in papillary muscle cells from *mdx* mice compared with WT (Sapp et al., 1996; Janssen et al., 2005). A plausible explanation for the compromised contractile response in *mdx* cardiac cells could be the altered intracellular Ca^2+^ regulation.

### WBPA Cardioprotection in *mdx* Mice With Cardiomyopathy

Extended lifespan in DMD patients has significantly increased recognition and development of cardiomyopathy. Although various curative and palliative therapeutic approaches, such as genetic and cellular, as well as pharmacologic therapies, are currently being investigated to treat DMD, these approaches are currently far from having a meaningful impact on the treatment of DMD patients (Bridges, 1986; Zincarelli et al., 2008; Popplewell et al., 2010; Wehling-Henricks et al., 2010; Schinkel et al., 2012; Zhang and Duan, 2012). Therefore, there are no effective treatments available for this devastating disease, and currently corticosteroids therapy still constitutes the primary treatment option for DMD patients, despite their serious side effects and a negligible effect in cardiomyopathy (Lu et al., 2005; Malik et al., 2012). Therefore, there is an increasing interest in developing DMD therapies that address dystrophin deficiency and target both cardiac and skeletal muscle. WBPA is an entirely different non-invasive and a non-pharmacologic approach that amplifies intracellular constitutive NO signaling (Wu et al., 2009; Uryash et al., 2015), provoking substantial benefit in excitable cells from *mdx* mice (Altamirano et al., 2014; Lopez et al., 2017, 2018). We have previously demonstrated that WBPA-elicited NO production alleviated the cardiomyopathy’s severity in mice lacking both dystrophin and utrophin protein expression (Lopez et al., 2017). In this study, we have extended previous findings presenting evidence that WBPA started at an age when the mice had an established cardiomyopathy provides cardioprotection in 15-month *mdx* mice.

In *mdx* mice, WBPA-elicited constitutive NO production decreased diastolic [Ca^2+^] and [Na^+^] overload. Although we do not precisely know the mechanisms by which WBPA improves Na^+^ and Ca^2+^ homeostasis in *mdx* cardiomyocytes, could be linked to the NO-anti-inflammatory and antioxidant effect (Uryash et al., 2015). It is well established that oxidative stress contributes to the cardiomyocyte damage in DMD (Jung et al., 2008). WBPA significantly increased the expression of endogenous antioxidants enzymes like glutathione peroxidase, catalase, and superoxide dismutase in the heart (Uryash et al., 2015). A reduction of oxidative stress may reduce the S-nitrosylation of the RyR2 diastolic SR Ca^2+^ leak (Fauconnier et al., 2010) and the TRPC channels resulting in a reduction of intracellular Ca^2+^ and Na^+^ overload. Furthermore, WBPA increases expression of p65 (major NF-κB subunit) and IκB-α (NF-κB repressor) in striated muscle (Altamirano et al., 2014). In this manuscript, we show that WBPA reduced ROS production in 15-month WT cardiomyocytes (Figure 3); those mice show an abnormal diastolic [Ca^2+^] [compared to 3-month WT (Mijares et al., 2014) and Supplementary Figure 1]. It is important to point out that WBPA did not modify [Ca^2+^]_d_ and ROS generation in WT 3-month cardiomyocytes (Supplementary Figure 1). There is a close connection between intracellular Ca^2+^ homeostasis and oxidative stress, and increasing evidence suggests close crosstalk between Ca^2+^ and ROS signaling systems, which fine-tunes cellular signaling networks (Gorlach et al., 2015).

Whole body periodic acceleration-elicited NO production induces upregulation of utrophin in *mdx* cardiomyocytes, which compensates for the absence of dystrophin in dystrophic muscle cells (Love et al., 1989). Utrophin is a fetal homolog of dystrophin expressed at the sarcolemma of immature striated muscle (Tome et al., 1994) and gradually is replaced by dystrophin in adult striated muscle cells (Karpati et al., 1993). The mechanisms by which NO induces upregulation of utrophin are not fully understood. One possibility is that WBPA-elicited production of NO would turn on the signal for fetal gene expression (Chaubourt et al., 1999). It is plausible to suggest that utrophin upregulation led to an increase of muscle function in dystrophic cardiomyocytes.

Here we demonstrated that a primary mechanism of action is through NOS mediated NO synthesis by showing that treatment of *mdx* mice with L-NAME, a non-selective inhibitor of NOS-synthase (Furfine et al., 1997), abolishes all beneficial effects WBPA on cardiomyocyte [Ca^2+^]_d_, and oxidative stress. Further studies are needed to study the downstream signaling involved in the cardioprotection caused by this essential bioregulatory molecule.

### Study Limitations

Despite the novel finding that WBPA mitigates some of the aberrations observed in *mdx* with established cardiomyopathy, some study limitations must be pointed out. We report that physiological enhancement of NO by WBPA induced upregulation of utrophin in *mdx* cardiomyocytes which elicited a partial correction of some of the observed cardiomyocyte pathological abnormalities. However, mechanism/s by which WBPA induced its effects were not explored. Another limitation of this study is that we demonstrated that an NO antagonist (L-NAME) blocks the cardioprotective effect of WBPA of lowering diastolic [Ca^2+^] and ROS production, suggesting that NO-mediates the beneficial effect; however, the undelaying mechanism was not determined, and the effects of L-NAME on utrophin expression was not studied. We have also only addressed diastolic Ca^2+^ dysfunction and not systolic, which would allow for additional estimation of the cardioprotection induced by WBPA. Finally, this study did not address a longer time of exposure (more than 1 h) or greater number of days (more than 90 days) of WBPA.

### Conclusion

Although the mechanism by which WBPA induced the observed cardioprotection has not been established yet, the observed benefits of WBPA could be related to the direct effect of NO on ionic channels (Meszaros et al., 1996; Zahradnikova et al., 1997), its anti-inflammatory and antioxidant effects (Uryash et al., 2015), its ability to enhance cellular repair (Anderson, 2000; Filippin et al., 2011), as well as its potential to upregulate the expression of structural plasma membrane proteins like utrophin (Altamirano et al., 2014). Whatever the precise mechanism, from the data presented here, we conclude that WBPA represents a promising therapy for preventing and/or treating DMD cardiomyopathy. This conclusion is supported by the fact that 3-month treatment with WBPA slows the development of cardiomyopathy in *mdx* 15-month old mice by normalizing intracellular ion dyshomeostasis, reducing intracellular ROS production and cell injury, which enhances contractile function and increases cell viability. WBPA as a therapeutic approach to treat DMD has some advantages compared with current available treatments; (1) WBPA is not a pharmacological approach, therefore no toxicology and preclinical pharmacokinetic studies are required before its use in patients; (2) is easily translated from being a research platform to patients; (3) It is non-invasive, non-stressful and able to be used even in those with physical limitations; (4) No side effects have been observed in experimental models of DMD and other diseases (Adams et al., 2014; Altamirano et al., 2014; Lopez et al., 2017, 2018; Sabater et al., 2019) as well as in various human clinical trials on non-DMD patients (Sackner et al., 2004; Kohler et al., 2007; Rokutanda et al., 2011; Sakaguchi et al., 2012; Serravite et al., 2014; Adams et al., 2020); (5) WBPA not only protects and improves function in cardiac cells but also has a similar effect in skeletal muscle (Altamirano et al., 2014) and in neurons (Lopez et al., 2018) all of which are affected in DMD.

## Data Availability Statement

The raw data supporting the conclusions of this article will be made available by the authors, without undue reservation.

## Ethics Statement

The animal study was reviewed and approved by the University of California, Davis (Protocol number: #17298) and Mount Sinai Medical Center (Protocol number: #14-22-A-04).

## Author Contributions

AU, AM, and JRL performed the research. AU, AM, EE, JA, and JRL analyzed the data. JRL wrote the manuscript. All authors contributed to the manuscript revision and read and approved the submitted version.

## Conflict of Interest

The authors declare that the research was conducted in the absence of any commercial or financial relationships that could be construed as a potential conflict of interest.

## Supplementary Material

Supplementary Figure 1 Effects of WBPA on [Ca^2+^]_d_ and ROS generation in cardiomyocytes from young WT and *mdx* mice. [Ca^2+^]_d_ and ROS production were evaluated in cardiomyocytes from 3-month WBPA-treated and untreated WT and *mdx* dystrophin-deficient mice. **(A)** [Ca^2+^]_d_ was 1.2-fold more elevated in *mdx* cardiomyocytes than WT. WBPA treatment normalized [Ca^2+^]_d_ in *mdx* cardiomyocytes compared to untreated WT (*p* = 0.08), with no effect on WT cardiomyocytes. **(B)** Intracellular ROS was 2.1-fold higher in *mdx* cardiomyocytes than WT. WBPA treatment normalized intracellular ROS production in *mdx* cardiomyocytes compared to untreated WT (*p* = 0.5), with no effect on WT. *[Ca^2+^]_d_ measurements*: Untreated and WBPA-treated WT, *N* = 3 mice per experimental condition, *n* = 17–18, respectively; Untreated and WBPA-treated *mdx N* = 3 mice, *n* = 17–19, respectively. Values are expressed as means ± S.D. One-way ANOVA with Tukey’s post-test, NS *p* > 0.05, ^∗∗∗^*p* ≤ 0.001. *ROS measurements*: Untreated and WBPA-treated WT, *N* = 3 mice per experimental conditions, *n* = 15. Untreated and WBPA-treated *mdx N* = 3 mice per experimental conditions, *n* = 19–20, respectively. Values are expressed as means ± S.D. One-way ANOVA with Tukey’s post-test, NS *p* > 0.05, ^∗∗∗^*p* ≤ 0.001.
