# Supplementary figures and images for: Cardioprotective Effect of Whole Body Periodic Acceleration in Dystrophic Phenotype mdx Rodent
Source: Front Physiol. 2021 May 4;12:658042. doi: 10.3389/fphys.2021.658042 (PMC8129504; doi:10.3389/fphys.2021.658042)

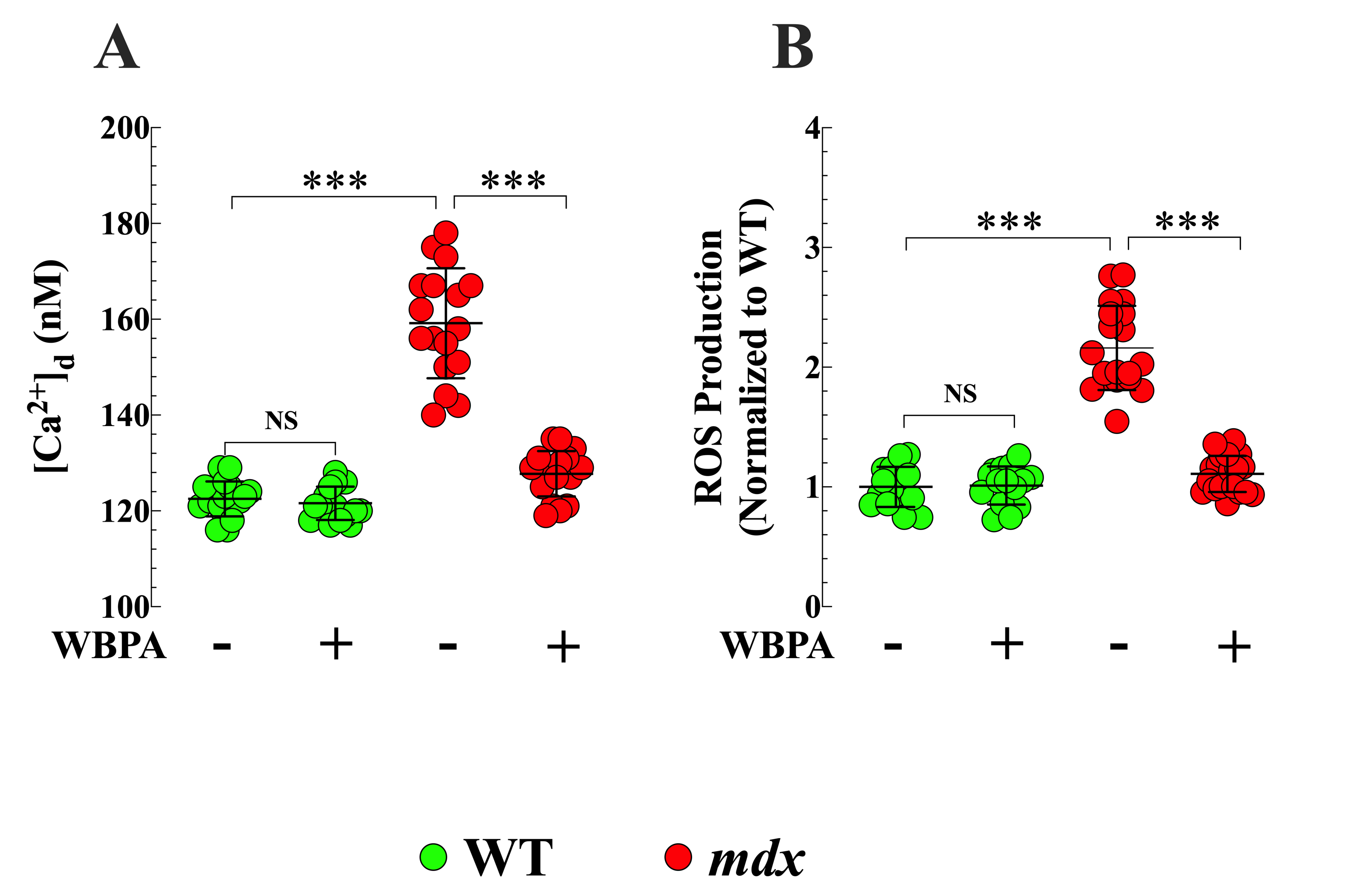

Supplement: Supplementary Figure 1 — Effects of WBPA on [Ca2+]d and ROS generation in cardiomyocytes from young WT and mdx mice. [Ca2+]d and ROS production were evaluated in cardiomyocytes from 3-month WBPA-treated and untreated WT and mdx dystrophin-deficient mice. (A) [Ca2+]d was 1.2-fold more elevated in mdx cardiomyocytes than WT. WBPA treatment normalized [Ca2+]d in mdx cardiomyocytes compared to untreated WT (p = 0.08), with no effect on WT cardiomyocytes. (B) Intracellular ROS was 2.1-fold higher in mdx cardiomyocytes than WT. WBPA treatment normalized intracellular ROS production in mdx cardiomyocytes compared to untreated WT (p = 0.5), with no effect on WT. [Ca2+]d measurements: Untreated and WBPA-treated WT, N = 3 mice per experimental condition, n = 17–18, respectively; Untreated and WBPA-treated mdx N = 3 mice, n = 17–19, respectively. Values are expressed as means ± S.D. One-way ANOVA with Tukey’s post-test, NS p > 0.05, ∗∗∗p ≤ 0.001. ROS measurements: Untreated and WBPA-treated WT, N = 3 mice per experimental conditions, n = 15. Untreated and WBPA-treated mdx N = 3 mice per experimental conditions, n = 19–20, respectively. Values are expressed as means ± S.D. One-way ANOVA with Tukey’s post-test, NS p > 0.05, ∗∗∗p ≤ 0.001. [file Image_1.TIFF]
